# The Psychedelic Policy Quagmire: Health, Law, Freedom, and Society

**DOI:** 10.1192/pb.bp.115.053165

**Published:** 2017-04

**Authors:** Adam G. Van Hagen

The resurgence of psychedelic research has opened up a new realm of possibilities in consciousness research. However, public policy surrounding the use of psychedelics has struggled to acknowledge that they may be effective therapeutic treatments or tools for enhancing self-awareness and exploring consciousness. Highlighting the need for redress, this anthology argues that current international public policy is not scientifically or culturally informed and is thus divorced from the empirical evidence that is supposed to inform its construction and implementation.

The book examines the complex policy issues surrounding psychedelic-based healing modalities and calls for an urgent shift in policy regulating the research and application of psychedelic substances. At its core, it is a scathing criticism of legal frameworks and regulatory policies that control the use of and research on psychedelics, and goes so far as to suggest that current structures and mechanisms impose a status quo of consciousness, thereby preventing people from fully enacting their right to freedom of religion, thought and conscience. At the very least, policy makers and ethicists need to give due attention to medical and psychotherapeutic research on psychedelics and the role they have in facilitating direct spiritual experiences. This includes acknowledging the transformative effect that experience may have on the self, as well as the right of all people to freedom of religion, thought and conscience.

Any book that rates these substances highly as a connection between the individual, society and the human race as a whole will find its detractors. That being said, *The Psychedelic Policy Quagmire* presents a strong case for the notion that psychedelics have transcended seemingly outdated legal, academic, cultural and spiritual paradigms. Although – by the editors' own admission – this volume is by no means definitive, it will undoubtedly prove to be a lightning rod in the academic community. With its focus on research and policy that maximise the benefits of the use of psychedelics, reduce the potential dangers of misuse and remove impediments to achieving these ends, it is inevitable that this book will be a catalyst for lively and robust debate. Recommended to academics and researchers in various fields, including psychology, psychiatry, anthropology and the arts, this work should challenge many long-held assumptions about these fascinating substances.

